# Effects of *Anma* massage therapy (Japanese massage) for gynecological cancer survivors: study protocol for a randomized controlled trial

**DOI:** 10.1186/1745-6215-14-233

**Published:** 2013-07-24

**Authors:** Nozomi Donoyama, Toyomi Satoh, Tetsutaro Hamano

**Affiliations:** 1 Department of Health, Faculty of Health Sciences, Tsukuba University of Technology, 4-12-7 Kasuga, Tsukuba, Ibaraki, 305-8521, Japan; 2 Department of Obstetrics and Gynecology, Faculty of Medicine, University of Tsukuba, 1-1-1 Tennoudai, Tsukuba, Ibaraki, 305-8575, Japan; 3 H-STAT Co Ltd, 5-11-14 Todoroki, Setagaya-ku, Tokyo, 158-0082, Japan

**Keywords:** *Anma* massage therapy, Body, Cancer survivors, Gynecological cancer, Health-related quality of life, Mind, Randomized controlled trial

## Abstract

**Background:**

Cancer patients and survivors regularly feel anxious about cancer recurrence or death, even after the conclusion of medical treatment, and they are often highly physiologically and psychologically stressed. Massage therapy is one of the most widely used complementary and alternative therapies used in the hope of alleviating such stress and physical and psychological complaints and to improve health-related quality of life. This randomized phase III, two-armed, parallel group, clinical trial was designed after obtaining positive findings in a preliminary study. The primary objective is to verify the effects of continuous Japanese massage therapy, referred to as *Anma* therapy, for cancer survivors. The secondary objective is to confirm the immediate effects of a single *Anma* massage session for cancer survivors.

**Methods/Design:**

Sixty cancer survivors older than 20 years of age who have had histologically confirmed uterine cervical, endometrial, ovarian, fallopian tube or peritoneal cancer in the past, but with no recurrence for more than 3 years since receiving standard medical treatment, are being recruited by gynecologists in medical facilities. In the coordinating office, they are randomly allocated to two groups (*n* = 30 each): an *Anma* massage group receiving a 40-min *Anma* massage session once weekly over a 2-month intervention period (total of eight *Anma* massage sessions) and a control group being followed by medical doctors and receiving no *Anma* massage sessions. The primary end point is the severity of physical subjective symptoms that cancer survivors report in daily life, assessed using a Visual Analogue Scale. Secondary end points are urine and saliva analyses, psychological condition and health-related quality-of-life scores as determined on the basis of a self-report questionnaire.

**Discussion:**

Using the evidence-based findings of this trial, medical professionals should be able to explain the benefits conferred by *Anma* massage to cancer survivors and provide higher-quality information to better inform patients regarding their decisions about whether to receive such therapy.

**Trial registration:**

This trial is registered with the UMIN Clinical Trials Registry as UMIN000009097.

## Background

Since 1981, cancer has been the leading cause of death in the Japanese population. In 2011, there were 357,305 cancer deaths (28.5% of all deaths), 210,000 of which were men and 140,000 were women, and the number of such deaths is increasing because of the aging of the population [1]. In addition, the incidence of cancer is increasing, exceeding 700,000 (410,000 men and 290,000 women) according to the latest data [2]. It is estimated that the yearly average number of cancer deaths and cancer incidence for the period 2025 to 2029 in Japan will be approximately 230,000 men and 160,000 women and 530,000 men and 390,000 women, respectively [3]. Both the number of cancer deaths [4] and cancer incidence [3] for men are expected to slow after 2015, whereas for women these figures, especially regarding the incidence of cancers of the oral cavity and pharynx, kidney and urinary tract, uterus, lung, pancreas and cervix are expected to continue increasing at the present rate [4]. It is also known that early detection and progressive treatment options have improved the prognosis of cancer patients and increased the number of cancer survivors in Japan [5], and accordingly interest has been shifting from radical treatment options toward ensuring a better quality of life (QoL) for patients to cope with the disease [6].

Cancer patients and survivors regularly feel anxious about cancer recurrence or death, even after the conclusion of medical treatment, and it is known that they are highly stressed, both physiologically and psychologically [7-9]. Although 65% of men living with cancer are older than 65 years of age, 68.2%, 72.8%, 68.7% and 70.6% of women with breast, uterine, thyroid and ovarian cancer, respectively, are younger than 65 years old [5]. Because the time women live with cancer is thought to be longer than that of men, it may be especially necessary to provide enhanced physical and mental care to female cancer survivors.

Numerous studies on massage for patients with cancer have been conducted in Western countries in recent years. The findings of many of these studies imply that massage therapy may help to improve the physical and psychological symptoms of cancer patients [10-13], and patients receiving massage therapy in a pilot study reported improvements in stress and QoL [14]. Massage is, in fact, one of the most commonly used complementary and alternative therapies for cancer [15].

Japanese massage therapy, known as *Anma* therapy (hereinafter *Anma* massage therapy), is one of the most popular complementary and alternative therapies in Japan. It has long been used with the aim of improving or alleviating physical and psychological symptoms. With high expectations for *Anma* massage therapy, we previously conducted a preliminary study to assess whether *Anma* massage therapy confers physical and psychological benefits in five cancer survivors who had undergone surgery for uterine cervical or endometrial cancer (stages Ia1 to IIa) [16]. All participants received *Anma* massage therapy consisting of eight 40-min *Anma* massage sessions over 4 wk. In regard to immediate changes in variables after a single *Anma* massage session, physical subjective symptoms assessed using a Visual Analogue Scale (VAS) were significantly improved, state anxiety score and salivary cortisol were lowered and secretory immunoglobulin A (s-IgA) was significantly increased. In addition, after seven continuous *Anma* massage sessions, VAS score, anxiety score and depression score were all lowered. On the basis of these preliminary findings, we designed the present randomized trial to verify the effects of *Anma* massage therapy compared with no *Anma* massage therapy. The primary objective of the trial is to verify the effects of continuous *Anma* massage therapy for cancer survivors. The secondary objective of the trial is to confirm the immediate physical and emotional effects of a single *Anma* massage session.

## Methods and design

### Study design and setting

This study is a randomized, two-armed, parallel group clinical trial. The coordinating office is at Tsukuba University of Technology, Japan. Gynecologists in other medical facilities introduce the coordinating office to patients who meet the eligibility criteria and confirm they do not meet any exclusion criteria.

When patients show interest in trial participation, the gynecologists send an introduction form to the coordinating office by facsimile. The coordinating office calls the introduced patient to decide the date on which to meet to explain the trial, and the patient visits the office to be given information verbally on the trial and reads the written trial description. After the meeting, the patient submits the consent form by hand, facsimile or mail upon deciding to register for the trial. Accrual started on 12 October 2012, and the term for patient registration for the trial is within 2 years from this date. Sixty participants are planned to be recruited.

### Ethical consideration and study registration

The study protocol was designed in accordance with the World Medical Association’s Helsinki Declaration [17] and the Ethics Guidelines for Clinical Research of the Ministry of Health, Labor, and Welfare of Japan [18]. This trial was approved by the Medical Ethics Committee of Tsukuba University of Technology, Japan, on 27 September 2012 (Approval No. 5) and was registered with the UMIN Clinical Trials Registry as application UMIN000009097 on 12 October 2012.

### Eligibility criteria

The inclusion criteria are (1) histologically confirmed uterine cervical, endometrial, ovarian, fallopian tube or peritoneal cancer in the past; (2) no recurrence of such cancer for more than 3 years since receiving standard medical treatment; (3) older than 20 years of age at the date of registration for the trial; (4) patient’s doctor deems the patient to be eligible for the trial; and (5) receipt of written informed consent from the patient to participate in the trial. The exclusion criteria are (1) active infections; (2) serious concurrent disease of the heart, liver or kidney, etc.; and (3) severe mental disorders.

### Randomization

**Figure 1:**
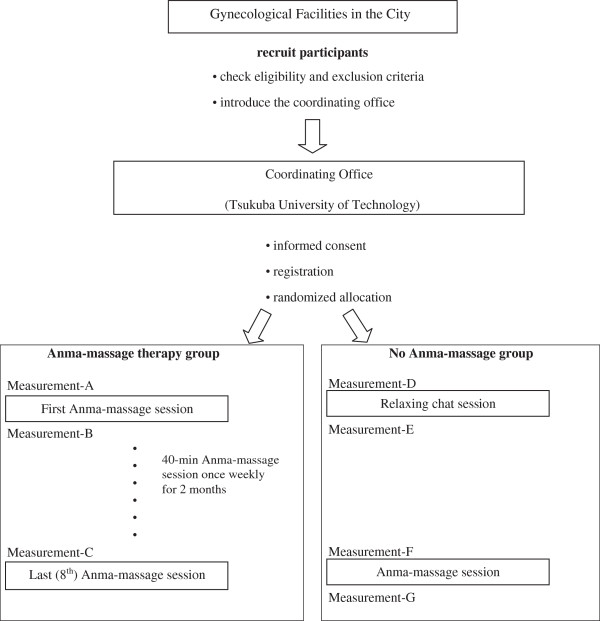
Trial Flow Diagram.

After finishing registration, the patients are allocated by block randomization by the coordinating office to continuous *Anma* massage therapy or no *Anma* massage therapy, except for a single *Anma* massage therapy intervention at the end of the trial period (Figure 1). Allocation adjustment factors are not set in the trial because of insufficient evidence at present regarding which factors affect the effectiveness of *Anma* massage therapy.

### Protocol treatment

#### Interventions

##### *Anma* massage therapy group

This group receives treatment by continuous *Anma* massage therapy according to the trial protocol. The protocol treatment is completed when the patient finishes receiving the final 40-min *Anma* massage session of a total of eight sessions given once weekly over a 2-month intervention period. *Anma* massage therapy techniques consist of standard versions of Japanese massage, mainly kneading with lesser amounts of stroking and pressing. *Anma* massage is performed through clothing, with the intensity of stimulation applied being within each person’s range of comfort. On a massage table, a full-body *Anma* massage excluding the face, head and abdomen is performed following the procedure described in detail in our previous studies [16,19,20], with a focus on the specific locations where patients want to improve physical symptoms. A therapist provides all massage sessions to avoid differences in technical capabilities. This therapist has a national massage practitioner license and over 20 years of experience and was the therapist involved in providing *Anma* massage treatment sessions in our previous studies [16,19,20].

##### No *Anma* massage therapy group

This control group is followed by their medical doctors as usual and do not receive the continuous *Anma* massage sessions. Patients visit the coordinating office and have a 40-min relaxing chat with a massage therapist while lying on a massage table, but without receiving a massage. Two months later they return to the office to receive a single 40-min *Anma* massage session.

### End points

The primary end point is the severity of subjective physical complaints that cancer survivors report in daily life as measured using a VAS. To assess the severity of subjective physical complaints, a sheet of paper (width 100 mm × height 40 mm) is given to the participant, and it is explained that the left edge of the paper represents no complaint and the right edge the most serious complaint that the participant can imagine. The participant records the seriousness of the degree of the subjective complaint at that time as a check mark on the paper. The length from the left edge to the check mark is measured and considered the VAS score.

The secondary end points of the study are as follows. (1) The score on the Profile of Mood State–Brief Japanese Version (POMS) questionnaire is used to assess immediate psychological effects. (2) The score on the Hospital Anxiety Depression Scale–Japanese version (HADS), a 14-item scale developed to assess anxiety and depression for particular cancer patients. (3) The score on the European Organization for Research and Treatment of Cancer QLQ-C30–Japanese version (EORTC QLQ-C30) scale, which was developed to assess health-related QoL, particularly in cancer patients. The subscales are global health status, functional scales (physical functioning, role functioning, emotional functioning, cognitive functioning and social functioning) and symptom scales (fatigue, nausea and vomiting, pain, dyspnea, insomnia, appetite loss, constipation, diarrhea and financial difficulties). (4) The score on the Measure of Adjustment to Cancer–Japanese version (MAC) questionnaire, because some studies [21,22] have reported a correlation between adjustment of cancer, QoL and mental health. Because some communication between the patient and massage therapist is commonly built into massage sessions, psychological adjustment to cancer (coping style to cancer) may change through such communication during continuous *Anma* massage therapy. (5) The level of 8-hydroxydeoxyguanosine and catecholamine (noradrenaline, adrenaline and dopamine) in urine is measured. (6) Levels of cortisol, chromogranin A and s-IgA in saliva are measured.

### Data collection

Data collection is performed on three occasions in the *Anma* massage group: before and after the first *Anma* massage session (measurements A and B) and before the last (eighth) *Anma* massage session (measurement C) (Figure 1). Data collection is conducted on four occasions in the control group: before and after the relaxing chat session (measurements D and E) and before and after the single *Anma* massage session (measurements F and G) at 2 months (Figure 1).

To assess the effects of continuous *Anma* massage sessions, changes in all end points between measurements A and C are compared with those between measurements D and F.

In addition, to confirm immediate changes following a single *Anma* massage session, which is the secondary objective of the trial, changes in VAS score, POMS score, and levels of 8-hydroxydeoxyguanosine, catecholamine (noradrenaline, adrenaline and dopamine), salivary cortisol, chromogranin A, and s-IgA between measurements A and B are compared with those between measurements D and E. Such changes between measurement F and measurement G are also considered as additional evidence to confirm the immediate effects of a single *Anma* massage session.

### Sample size determination

In our preliminary study [16], the mean VAS score before the first *Anma* massage session in cancer survivors was 40.4, which was reduced to 19.6 before the last (eighth) *Anma* massage session. The mean VAS score difference during 2 months was −20.8 (standard deviation (SD) 19.6). We assume for the no *Anma* massage group that the mean difference will be unchanged (that is, zero) and that its SD will be the same as that of the *Anma* massage group (that is, 19.6). To test these differences between the two groups, with a 5% type I error rate and 90% power, a sample size of 14 patients per group is required. For sensitivity analysis, we also consider the following situations: the mean VAS score differences in the no–*Anma* massage group will be −5, –7.5 and −10, in which case sample sizes of 24, 33 and 49 patients per group, respectively, are required for 90% power, and 18, 25 and 37 patients per group, respectively, are required for 80% power. Therefore, the planned sample size will be 30 participants per group (60 participants in total). A sample size of 30 patients per group will also account for possible data loss or dropouts.

### Statistical analysis

Analysis of pretreatment characteristics and efficacy analysis will be performed according to the modified intention-to-treat principle and will include all participants who receive at least one *Anma* massage session in the *Anma* massage therapy group and one relaxing chat in the no–*Anma* massage group. If necessary, we might also perform sensitivity analyses, adding all eligible patients who are introduced by medical doctors to the coordinating office but are not registered.

The primary end point is VAS score improvement over the 2-month study period. For primary analysis, we will use the analysis of covariance to compare the mean changes in VAS score over the 2 months between the *Anma* massage group and the no–*Anma* massage group, adjusting for the baseline VAS score and age. If we find other significant prognostic factors, we will use them as additional adjusting factors. We will also use a two-sample *t*-test with Satterthwaite’s approximation to compare the mean changes. We will also test the mean changes in VAS score for each group using a paired *t*-test. For these mean VAS score changes and their differences, we will also calculate two-sided 95% confidence intervals (CIs) to evaluate the clinical effects.

For the secondary end points, we will not consider multiplicity issues. In all analyses, categorical variables will be described in terms of frequency and percentage. The distributions of continuous variables will be described using means, SDs, medians, and minimum and maximum values. A two-sample *t*-test or paired *t*-test will be used to detect differences in continuous variables. Pearson’s χ^2^ test with continuity correction will be used to test differences in categorical variables. If these data are found not to be normally distributed, we will use in their place the Wilcoxon signed-rank test or the Mann-Whitney *U* test, respectively. All reported *P* values will be two-sided. All significance levels will be set at 0.05, and reported CIs will be 95%. In principle, we will use the available-case analysis for missing outcomes. SAS statistical software (version 9.3 or later; SAS Institute, Cary, NC, USA) will be used for all analyses.

## Discussion

This trial is considered to correspond to a phase III study because of the preliminary study undertaking and because the randomized confirmatory trial has significance for the primary end point set at 5%, addressing the effects of longer-term intervention. This trial will employ adequate methods to reduce bias, such as randomization, a large subject population and analysis according to the intent-to-treat principle.

Regardless of whether the results of this trial are positive or negative, in the current situation, where benefits conferred to the body and mind by *Anma* massage therapy for cancer survivors are controversial, the findings of this trial will help to provide an answer to this controversy and will be reflected in the guidelines for cancer care. At present, patients decide of their own will whether they receive *Anma* massage therapy. Using the evidence-based findings of this trial, medical professionals should be able to explain the benefits conferred by *Anma* massage to cancer survivors and provide higher-quality information to better inform patients in their decisions about whether to receive such therapy.

## Trial status

Start date: October 12, 2012

Expected end date: October 11, 2014

Expected publication date: March 31, 2015

Status at time of submission of this article: Recruiting

Funder: The present study is supported by a Grant-in-Aid (No. 22531058) for Scientific Research from the Ministry of Education, Culture, Sports, Science and Technology, Japan, 2010–2014. The principal investigator is Nozomi Donoyama.

## Abbreviations

Anma massage therapy: Japanese massage/*Anma* therapy; CI: Confidence interval; EORTC QLQ-C30: European Organization for Research and Treatment of Cancer QLQ-C30; HADS: Hospital Anxiety Depression Scale; MAC: Measure of Adjustment to Cancer; POMS: Profile of mood state–brief Japanese version; QoL: Quality of life; s-IgA: secretory immunoglobulin A; VAS: Visual analogue scale.

## Competing interests

All authors declare that they have no competing interests.

## Authors’ contributions

DN conceived the trial. DN, ST and HT designed the trial. DN drafted the protocol. ST and HT are supervising data management and patient registration. HT is responsible for statistical analysis. DN wrote the manuscript. All authors read and approved the final manuscript.
